# Value-conscious leadership actions in developing a health-promoting work environment

**DOI:** 10.1177/09697330251366615

**Published:** 2025-09-08

**Authors:** Diako Morvati, Rita Solbakken, Jonas Vaag, Yvonne Hilli

**Affiliations:** 1786 Nord University; 1786 Nord University; 639938 University of Inland Norway; 1786 Nord University

**Keywords:** Caring science, health promotion, hermeneutics, leadership, nursing, work environment

## Abstract

**Background:**

Nurse leaders play a vital role in fostering a health-promoting work environment. Despite the increasing recognition of the importance of their roles, studies focusing specifically on the actions they employ to foster such environments remain limited.

**Research aim:**

The aim of this study is to explore and enhance understanding of the actions nurse leaders employ to develop a health-promoting work environment.

**Research design:**

This study used a qualitative design inspired by Gadamer’s philosophical hermeneutics. Four semi-structured focus group interviews were conducted between August and December 2024. The data were analyzed using reflexive thematic analysis.

**Participants and research context:**

Fourteen nurse leaders from hospitals and nursing homes in northern Norway participated in this study.

**Ethical considerations:**

The study was approved by the Norwegian Agency for Shared Services in Education and Research. Both oral and written informed consent was obtained from the participants.

**Findings:**

The actions employed by leaders to develop a health-promoting work environment are imbued with an overarching theme: “value-conscious leadership in action,” and four interwoven themes: (1) promoting justice by leading with equity and flexibility, (2) promoting relationships by cultivating an inclusive community, (3) respecting employees by fostering their empowerment and autonomy, and (4) facilitating professional growth by promoting a learning environment.

**Conclusions:**

The basic values should be articulated, consciously integrated, and embodied through the nurse leader’s actions, choices, and way of being. To implement the basic values, it is important to establish supportive networks for leaders and to conduct regular ethical reflections together with employees in the workplace.

## Introduction

Nurse leaders play a vital role in fostering a health-promoting work environment (HPWE),^1–4^ which is crucial for nurses’ job satisfaction, reducing turnover and burnout, improving retention of healthcare professionals, and enhancing patient safety and quality of care.^1–3,5^ Despite increasing recognition of the importance of nurse leaders’ roles, studies focusing specifically on the actions they take to develop an HPWE remain limited.^6,7^ This study seeks to address the knowledge gap by conducting group interviews with Norwegian nurse leaders, with the aim of exploring how they articulate and reflect upon their own actions in the development of an HPWE.

## Background

Leadership in nursing involves continuous decision making and taking action.^2,6,8^ Nurse leaders’ actions are based on their values and priorities, and the way leaders choose to act can influence the work environment.^6,9^ In this study, “nurse leader” refers to a leader with a nursing background who holds a formal mid-level leadership position, works closely with patients and personnel, and is responsible for personnel, finances, and patient care in clinical practice.

Earlier studies have shown that various forms of relational-based leadership, such as authentic leadership,^10^ servant leadership,^11^ and transformational leadership,^12^ have guided leaders in promoting nursing staff well-being and job satisfaction. However, these leadership theories have mostly been drawn from and adopted outside the contexts of the nursing and caring sciences.^13^

The theoretical perspective in this study is based on the theory of caritative leadership, developed from Eriksson’s caring sciences theory.^13,14^ Caritative leadership stems from an ethos of love for humanity, intending to promote health, alleviate suffering, and respect human dignity.^13^ In the caring science tradition, ethos and ethics belong together. Ethos represents a way of being, an attitude that fosters a sense of responsibility toward fellow human beings.^14^ Ethos is the person's basic values that promote the good life, where a person feels metaphorically “at home.” The home as ethos comprises three dimensions. The innermost dimension is imbued with a person's basic values or ethos. The middle dimension encompasses a person's manner of being an ethical code of conduct, all shaped by the ethos and reflected in the third, physical, and outermost dimensions, where persons meet, act, and interact.^15^ Leaders need to be aware of their basic values and ensure that these values are reflected in their actions and interactions in the workplace.^13,16^

In this study, action is understood according to Aristotle’s concept of praxis (action), which emphasizes moral and ethical actions aimed at achieving the good life (eudaimonia) through virtue and practical wisdom (phronesis). Phronesis involves action based on values aimed at human good. Aristotle distinguishes praxis from both theoretical knowledge (theoria), which involves intellectual contemplation, and productive activity (poiesis), which focuses on creating external results. Praxis, then, refers to an action that has a goal in itself, with that goal residing within the actions themselves. In other words, it is driven by the aim of living well and contributing to the community, guided by one’s moral virtues and ethos.^17^

A recent meta-ethnography metaphorically describes am HPWE as a tree with three key aspects: core values (roots), value-conscious leadership (trunk), and safe working conditions (fertile soil) encompassing both physical and administrative aspects. Value-conscious leadership, as the trunk, serves as a vital link between the roots (core values) and the branches (employees and their tasks), ensuring a cohesive and thriving work environment.^6^ In addition, earlier studies emphasize the importance of leadership qualities such as supportiveness, authenticity, visibility, responsiveness, and accessibility in creating an HPWE.^16,18,19^ Moreover, studies have highlighted that nurse leaders contribute to HPWE by facilitating open communication, fostering learning and professional development, providing constructive feedback, encouraging engagement, offering mentorship, and promoting nurses’ autonomy.^3,20^ In earlier studies, nurse leaders emphasized the importance of maintaining appropriate staffing levels and aligning nurses’ competencies with patients’ needs in developing of HPWE.^21,22^ However, they also underlined that they continue to face ongoing challenges in balancing leadership responsibilities with the increasing demands for efficiency, standardization, and cost-saving management.^8,16^ Since the rise of New Public Management (NPM) in the 1980s, nurse leaders in many countries, including Norway, have faced growing pressure to prioritize productivity and cost-effectiveness over care values in their leadership praxis, leading to leaders experiencing a sense of living in two different worlds.^9,16^ The tension between different values can affect the work environment and increase stress levels among nurses, potentially leading to negative consequences for their health. Therefore, nurse leaders need to be able to balance competing values in their leadership practice and actions, as these have significant impacts on employees’ health and job satisfaction.^23^

## Research aim

The aim of this study is to explore and enhance understanding of the actions nurse leaders employ to develop a health-promoting work environment.

### Research design

This study used a qualitative design inspired by Gadamer’s philosophical hermeneutics.^24^ According to Gadamer, understanding is intrinsically linked to praxis—human action and lived experience. Understanding depends on our prejudices or pre-understanding and takes shape through a dialectical movement known as the hermeneutic circle—that is, the interplay between the whole and the parts, between what we interpret, the context, and our pre-understanding. This movement occurs, among other things, through open dialogue, where the participants engage in a shared exploration of the subject matter, or “die Sache,” in this case, an HPWE. The goal of dialogue is not to convince others to adopt a particular perspective but rather for both parties to collaboratively explore insights and achieve mutual understanding.^24^

### Participants and research context

Fourteen nurse leaders from hospitals and nursing homes in northern Norway participated in this study. A purposive sampling strategy was employed, with inclusion criteria stipulating that participants must (i) be a nurse leader in a formal mid-level leadership position, (ii) possess at least a bachelor’s degree in nursing, (iii) have a minimum of 1 year of leadership experience, and (iv) work full-time (100%) in a leadership role, considering that some nurse leaders may have reduced or shared positions. Participation comprised 11 women and 3 men, with an age range of 32–57. Most of the participants had additional education in leadership (*n* = 11), while one had a specialization in geriatric nursing, another in intensive care nursing, and one had no further education. Given the limited participation of only three men and the relatively small size of hospitals and municipalities in northern Norway, demographic characteristics are presented cautiously to ensure anonymity (Table 1).

**Table 1.** An overview of the characteristics of participants and contexts.

Focus group	Number of participants	Age (mean)	Leadership experience: (Mean)	Context
Focus group 1	3	37.3	7.6	Nursing Homes, Surgical Unit
Focus group 2	4	47.3	9.2	Nursing Homes, Physical Medicine & Rehabilitation, Medical Unit
Focus group 3	3	43.3	6.6	Nursing Home, Medical Unit
Focus group 4	4	51.5	9.7	Nursing Home, Medical Unit Intensive Care Unit

### Data collection

The data were collected through four digital semi-structured focus group interviews conducted using Microsoft Teams between August and December 2024.^25^ Focus group interviews were chosen for their ability to foster group dynamics and facilitate dialogue and discussion. The interactive nature of focus groups allows participants to build upon each other’s responses, leading to richer and more nuanced insights in comparison to individual interviews.^25^ Each focus group was conducted during working hours, composed of three to four participants, with each interview lasting approximately 2 hours, a duration deemed sufficient for digital meetings.^26^ An interview guide was developed based on the authors’ pre-understanding, which is reflected in the background through the presentation of previous studies and theoretical perspectives. The interviews were open and dialogical in nature, encouraging participants to share their experiences. They began with the following open-ended question: “Can you please describe a health-promoting work environment?” Follow-up questions were based on participants’ responses, such as “Can you share your experiences on how you act to develop such an environment?” The first author, the moderator, and the last, the co-moderator, participated in the focus group interviews. The determination of sample size was based on the concept of information power.^27^ This approach emphasizes obtaining sufficient and relevant information to provide meaningful qualitative insights, rather than aiming to reach a saturation point, which can be challenging in focus group dialogues, due to the dynamic and dialectical nature of the conversations. After conducting four focus group interviews, it was considered that no new information emerged, and the phenomenon was deepened through various aspects and nuances. The interviews were audio-recorded and transcribed verbatim immediately after each session.

### Data analysis

The data were analyzed and interpreted using reflexive thematic analysis (RTA), as described by Braun and Clarke.^28,29^ The first author transcribed and analyzed the interviews, engaging in frequent discussions and collaboration with the other authors. Following several rounds of discussion, consensus was reached on the analysis and findings. The data were organized manually using Microsoft Word. The interpretation process consisted of six steps: (1) The data were first familiarized by listening to audio recordings and repeatedly reading the transcribed interviews to an overall understanding. Initial thoughts and reflections were noted during this process. (2) Next, meaningful units and patterns in the data were identified and coded flexibly and inductively, with both conceptual and semantic coding focusing on aspects relevant to the research questions. (3) These codes were then grouped into potential sub-themes by examining the relationships and patterns among them. (4) The sub-themes were reviewed for coherence and nuance and then refined to highlight their core meanings. They were subsequently grouped into four main themes. (5) Each theme was clearly described and named, with a reflexive articulation of its core meaning and significance. (6) Finally, the analysis was written up, with themes illustrated using selected participant quotes (Supplemental File 1). The presentation of the findings was not merely descriptive but interpretative, integrating the authors’ interpretation and understanding. The interpretation process was therefore not linear; rather, it was dialectical, with frequent movement back and forth between phases to ensure a deeper understanding of the leaders’ experiences.^28,29^

### Ethical considerations

This study adheres to the research ethics guidelines of the Norwegian National Research Ethics Committee for Medicine and Health Sciences The Norwegian National Research Ethics Committees,^30^ which are based on the principles of The World Medical Association.^31^ This study was approved by the Norwegian Agency for Shared Services in Education and Research (SIKT, Ref. No. 389393). All participants received both verbal and written information about the study and signed an informed consent form before the interviews. All data were immediately anonymized by replacing personal and group information with a code (Groups 1–4 and P1–P14). The audio files were deleted after transcription.

## Findings

**Figure 1.**
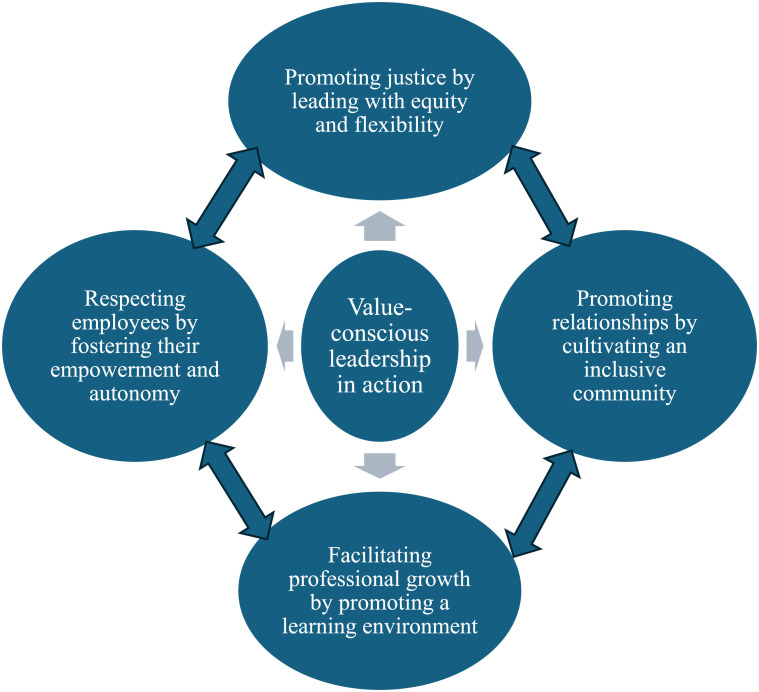
Value-conscious leadership actions in developing a health-promoting work environment.

The actions employed by leaders to develop an HPWE are imbued with an overarching theme: “value-conscious leadership in action,” and four interwoven themes: (1) promoting justice by leading with equity and flexibility, (2) promoting relationships by cultivating an inclusive community, (3) respecting employees by fostering their empowerment and autonomy, and (4) facilitating professional growth by promoting a learning environment (Figure 1).

### Promoting justice by leading with equity and flexibility

According to the nurse leaders, acting justly is central to developing an HPWE. Justice involves ensuring fairness by providing individualized resources and support based on the individual’s needs and prerequisites, ensuring that everyone has a fair chance of succeeding. This approach is described as essential for building trust and fostering a sense of safety among employees:

I believe that justice is one of the most important grounds for a health-promoting work environment... If someone receives special benefits without it being clear why, it can lead to unrest and unsafety among others (Group 4—P13).

In this context, acting justly also entails transparency and openness in the decisions and adaptations made for individual employees. The leaders described justice not only as equal treatment but also as a balancing act between upholding equal principles for all while also demonstrating flexibility in addressing individual prerequisites and circumstances. This means acting with equity:

The ability to balance as a leader involves responsibility for laws, finances, and other hard values. At the same time, a leader must also balance the care for employees (Group 2—P5).

Therefore, to choose the just way to act, the leaders need to be conscious of managerial regulation, rules, and directions on one hand, and humanistic values on the other. A leader expresses the importance of values in this way:

Values are like the ground of a house... if the ground of a house begins to crack, the entire house will collapse (Group 4—P14).

A prerequisite for acting justly is being genuinely interested in the people you lead as unique individuals by showing consideration for their problems, challenges, and difficulties. This can, for example, include facilitating flexible shift work, ensuring adequate staffing at the workplace, or providing extra resources to reduce the workload:

It is also about trying to adapt as well as possible to each individual; we humans have different interests and resources… different challenges and potential illnesses (Group 1—P1).

Being a just leader, therefore, means more than just following set schedules and rules; it also requires an understanding of interpersonal relationships and the ability to adapt to individual needs to promote employee health and well-being.

### Fostering relationships by cultivating an inclusive community

As the leaders emphasized the importance of interpersonal relationships, they underscored the importance of spending time with employees and being visibly engaged in daily interactions, as this is crucial for fostering strong workplace relationships. By actively participating in daily work assignments and acting as role models, embodying the leaders’ values through their actions, they demonstrate their commitment to supporting others and fostering a sense of teamwork:

I walk through the ward, listening and observing the atmosphere...I contribute in challenging situations and usually wear my white uniform … I think it creates a sense of closeness (Group 4—P14).

Through active participation, the leaders strive to cultivate a spirit of collaboration, mutual respect, and community, demonstrating their role as integral members of the team. By doing so, they not only set a tone for the team but also reinforce an environment where employees feel supported and valued. Furthermore, the leaders highlighted the importance of creating spaces for a support group characterized by openness, tolerance, and respect for diversity. This space would provide employees with the opportunity to express and exchange their experiences, concerns, challenges, and differences without fear of judgment or negative consequences:

It must be acceptable to be different … It is important that there is space to be who you are and that you are accepted for it (Group 2—P6).

Such a space is especially vital after incidents involving conflicts, such as patient violence and threats, as it allows employees to process emotional stress and provide mutual support during difficult times. While collegial support through a supportive group is crucial for handling challenges and emotional stress, the leaders emphasized the importance of organizing activities and social gatherings outside of working hours, as this provides employees with the opportunity to meet in a different context, without work uniforms, and get to know each other as whole individuals and friends. Humor is especially emphasized as a crucial element of such gatherings, as it helps foster a pleasant and friendly environment. Therefore, the leaders view it as their responsibility to facilitate the creation of a social committee that organizes events such as Friday lunches, café meet-ups, outdoor activities, trips, and celebrations of employees’ birthdays and special occasions:

We have selected a group of employees who have been assigned the responsibility of organizing regular social gatherings, either with meals or activities (Group 1—P3).

### Respecting employees by fostering their empowerment and autonomy

According to the leaders’ experiences, an HPWE is characterized by empowered employees who take responsibility and act autonomously. Empowering employees is closely linked to respect, as it involves recognizing their abilities, trusting their judgment, and providing them with the autonomy to contribute to decision making and influence both their workday and decision-making processes:

We want to include people, those on the floor who know where the shoe pinches...I want the employees to be able to participate in what's happening in the organization as much as possible (Group 2—P6).

By involving employees in decision-making processes, leaders strive to cultivate trust and a sense of agency, empowering them to influence their daily work conditions. This not only builds confidence but also fosters a sense of value and mutual respect within the workplace. The leaders mentioned several actions, such as anonymous work environment surveys and colleague conversations, that provide employees with opportunities to share their perspectives. However, they also acknowledged that these actions are insufficient due to their infrequency:

But a colleague conversation is once a year, so if an employee only gets to participate once a year, it's not very often (Group 3—P8).

Therefore, more frequent meetings could provide employees with better opportunities to influence their workdays. One key point emphasized by the leaders was the importance of encouraging employees to think critically, address challenges in the workplace, and, most importantly, take their thoughts, concerns, and suggestions seriously and ensure that these are not left unaddressed without follow-up actions:

Suggestions, changes, and perhaps what might feel like criticism, but we don't call it criticism... we call it improvement all the way…we incorporate it into plans and schedules (Group 4—P13).

Taking employees’ opinions seriously means recognizing them as unique and valuable individuals. By recognizing and valuing employees’ efforts and engagement, the leaders contribute to strengthening employees’ confidence in taking responsibility and making independent decisions:

I believe that recognizing what they do... in a way, giving them feedback when they've done something, praising them (Group 1—P3).

By providing feedback and valuing employees’ uniqueness, along with their unique skills and competencies, the leaders foster a sense of mastery and confidence in taking responsibility. According to the leaders, responsibility involves taking ownership of tasks and processes within their areas of expertise, particularly concerning activities related to organizational improvements or system changes that directly impact their workday:

…If you give the employees responsibility and get them to present something to you, then they can also influence work situations (Group 3—P8).

At the same time, the leaders emphasized that a prerequisite for strengthening employees’ ability to take responsibility and act autonomously is ensuring they have access to essential resources, such as competence and information, which enable them to make informed decisions and take action:

I also strongly believe in sending out information to everyone...information that employees should know about (Group 3—P10).

### Facilitating professional growth by promoting a learning environment

Based on the leaders’ experiences, strengthening employees’ competence is essential for fostering a sense of mastery, increasing their autonomy, and enabling them to realize their potential, which in turn is crucial for professional growth and the development of an HPWE. Facilitating professional growth involves fostering a learning environment that considers employees’ individual learning interests and needs. It is crucial that these are aligned with patient needs and tailored to the specific demands of their work environment and tasks. Conducting a thorough assessment of learning needs was emphasized as a crucial first step in initiating the learning process. Furthermore, organizing employees into various reflection groups based on shared interests was mentioned as a useful action for fostering engagement in professional development:

We have divided into different groups, and it is also adapted according to individual interests. If someone wants to work with stroke patients or palliative care, we can facilitate that (Group 2—P6).

Ensuring that the right competencies are in the right place was further highlighted by the leaders as crucial for facilitating employee growth. They mentioned that, in response to the ongoing nursing shortage, they have started exploring alternative approaches to optimize the use of nurses’ competencies. For instance, they pointed out that role clarity, along with clear job descriptions, responsibilities, and task-sharing, are essential strategies for achieving this goal. Task sharing was described as delegating specific nursing tasks and responsibilities to other team members, such as healthcare workers or nursing assistants, based on their competencies, qualifications, and the patient’s situation and needs:

Here with us, after implementing the “optimal use of competence” method, we started evaluating which tasks nurses currently perform that could be handled by other qualified personnel (Group 1—P3).

To ensure continuity and ongoing learning, the leaders mentioned several actions, such as having a professional development nurse for all employees and implementing a mentorship program specifically for newly graduated nurses. The professional development nurse, in collaboration with leaders, will organize various courses, arrange for employees to attend specialized training programs, motivate employees to pursue further education, and train facilitators to focus on areas that are crucial for professional development and improving practice:

I have a close collaboration with the professional development nurse, where we go through various forms to map each employee's competencies…so based on their needs, we provide training for everyone (Group 1—P2).

Although leaders place strong emphasis on motivating employees to participate in courses or pursue further education, they acknowledge that this is often challenging due to the limited funding available to support employees’ participation in these programs:

But we don't get any money for competence.... If I have to send someone to a conference for three days, I'm missing a nurse for three days (Group 2—P7).

## Discussion

This study aimed to explore and enhance understanding of the actions nurse leaders employ to develop an HPWE. Nurse leaders highlighted their commitment to fostering an HPWE through actions that promote justice, strengthen relationships, respect, and empower staff, and encourage continuous learning. Justice in nursing leadership has been emphasized as an important aspect in the development of an HPWE^32,33^ and in reducing psychological and emotional strain.^34^ Justice is commonly understood as equality for all.^35^ However, the leaders in this study described justice as extending beyond mere equality and shifting the focus to equity. This concept acknowledges that treating everyone in the same way is not always fair. Instead, it recognizes that people have different needs, circumstances, and starting points, so they may require different levels of support to achieve the same opportunities.^35–37^ According to Aristotle, equity (epieikeia) is a higher form of justice, as it considers concrete situations in which a strict application of the law would lead to unreasonable or unjust outcomes.^17^

In other words, justice-promoting action is not an abstract principle but an exercise of phronesis—practical wisdom. This means that leaders must be conscious of their ethos and virtues, along with sensitivity to the context and individual uniqueness.^17,38^ Ethos and phronesis are closely linked, as individuals with profound practical wisdom tend to make decisions and act in alignment with their values.^14,15^ Value-conscious leadership acts as a motivator and tone-setter, influencing the atmosphere in the environment and realizing the basic human values in the work environment.^6^ When leaders act in harmony with their ethos, they are internally driven to foster an environment where all employees feel metaphorically “at home,” a space where everyone is treated fairly and respectfully.^6,8,16^

As in earlier studies, our study shows that by actively engaging in daily work assignments, acting as role models, and embodying values in their actions, leaders can cultivate a spirit of collaboration, mutual respect, and a supportive and inclusive community.^6,13,16,18,39^ A supportive and inclusive community is characterized by an environment in which employees’ uniqueness and differences are respected, allowing them to express their experiences, concerns, and challenges freely without fear of judgment. Earlier studies have shown that openness and avoiding judgment cultivate an honest and welcoming atmosphere, providing a foundation for employees to flourish and thrive both personally and professionally.^6,40^ On the one hand, belonging to such a community leads to the creation of a friendly, home-like atmosphere that, in turn, enhances employees’ health and well-being.^6,40^ On the other hand, feelings of not belonging to a community may lead to alienation, potentially resulting in a sense of homelessness and suffering.^6^ Nurses have previously reported being exposed to psychological and emotional stress as part of their job.^41^ The leaders in this study were aware of this and therefore emphasized the importance of establishing a support group where employees can share their emotional experiences. Moreover, previous studies have shown that supportive, empowering, and fair leadership, in itself, can reduce the psychological burden of emotional dissonance.^6,34^

This study, consistent with previous research, emphasized the leader’s role in empowering employees through decision-making involvement, providing opportunities to influence daily work conditions, and assigning responsibilities that match their competence, thereby fostering ownership, confidence, autonomy, and participation—key elements in developing an HPWE.^3,4,6,18,20^ An important point to note is that the opportunity to discuss salary was not identified in this study as a significant factor influencing employees’ work conditions. However, previous studies have emphasized the importance of salary as one of the key factors that indirectly affect the work environment, job satisfaction, and employees’ health.^4,42,43^

A key point emphasized by the leaders, not widely explored in previous nursing leadership studies, is the importance of encouraging critical thinking and taking employees’ concerns seriously, with appropriate follow-up actions. This reflects a moral responsibility, ensuring employees feel valued, recognized, heard, and respected.^6,13,14^ Furthermore, our findings underscore that empowerment involves more than simply delegating responsibility; it requires providing the necessary support and resources, such as information, competencies, and access to ongoing education and training opportunities, as highlighted in previous studies.^4,6,18,22^ Role clarity and task-sharing were highlighted by leaders as important actions for developing nursing competence. However, studies indicate that delegating nursing tasks to other healthcare workers can lead to uncertainty and a perceived loss of control, as nurses may question the quality of task execution.^44,45^ Therefore, for the successful delegation of nursing tasks, it is crucial to enhance awareness and knowledge regarding the competencies and skills required by other healthcare workers to properly execute delegated tasks.^45^

## Methodological considerations

This study adheres to the Standards for Reporting Qualitative Research^46^ to enhance trustworthiness and transparency (Supplemental File 2). A potential limitation in hermeneutic studies is the researchers’ insufficient awareness of their pre-understanding, which may influence data interpretation.^47^ Our pre-understanding included our professional backgrounds in nursing education, nursing leadership, and organizational psychology, as well as prior knowledge and understanding of the topic, as described in the background section. This pre-understanding was continuously reflected upon and critically examined throughout the research process, as we were actively engaged in all phases of the study. Feedback and peer discussions challenged our pre-understanding and encouraged critical reflection. In addition, direct participant quotes were included to enhance the credibility and trustworthiness of the findings. The participants were employed across diverse contexts, including nursing homes and various hospital departments, which may have influenced the findings. However, during the group dialogue, the participants reflected on their own actions, values, and intentions. What emerged was not a contrast between contexts but rather a shared set of core principles and leadership actions that seemed to transcend the specific contexts. Another limitation of this study is that conducting the interviews digitally may have impacted the group dynamics. In a physical setting, facial expressions and body language could have influenced how the participants engaged with and responded to the questions.^48^ To mitigate this, all interviews were conducted with cameras on, enabling both the moderator and co-moderator to observe facial expressions, gestures, tone of voice, pauses, and interaction patterns, and to use follow-up questions to clarify meaning. Due to Norway’s geography and vast distances, nurse leaders in remote areas are often underrepresented in research. The use of digital platforms addresses this by enabling broader participation and mitigating this limitation. A further potential limitation of the study is that only 3 of the 14 participants were male, which may have influenced the findings.

## Conclusions

This study highlights the importance of nurse leaders’ consciousness of basic ethical values such as respect, justice, dignity, and relationships in the development of an HPWE. The findings demonstrate how these values can be evidenced and made visible in leaders’ actions. The basic values should be articulated, consciously integrated, and embodied through the nurse leader’s actions, choices, and way of being. To implement the basic values, it is important to establish supportive networks for leaders and to conduct regular ethical reflections together with employees in the workplace.

However, in today’s context characterized by time constraints, economic pressures, and administrative demands, facilitating time and space for reflection on basic values can be challenging. Therefore, it is essential that decision-makers recognize the importance of these basic values in leadership and actively support leaders by providing the necessary room for reflection and fostering their autonomy to act in accordance with these values. Supporting ethical reflection and value-based leadership is not only about promoting leaders’ moral integrity but also about encouraging meaningful work, engagement, and ethical practice among staff, which enhances their job satisfaction and consequently improves the quality of patient care. Future research should focus on critically exploring the structural, institutional, and discursive barriers and challenges that prevent nurse leaders from applying value-based actions in developing an HPWE.

## Supplemental Material

Supplemental material - Value-conscious leadership actions in developing a health-promoting work environment Supplemental material for Value-conscious leadership actions in developing a health-promoting work environment by Diako Morvati, Rita Solbakken, Jonas Vaag, and Yvonne Hilli in Nursing Ethics.

Supplemental material - Value-conscious leadership actions in developing a health-promoting work environment Supplemental material for Value-conscious leadership actions in developing a health-promoting work environment by Diako Morvati, Rita Solbakken, Jonas Vaag, and Yvonne Hilli in Nursing Ethics.

## Data Availability

The data that support the findings of this study are available from the corresponding author upon reasonable request.*
