# Supplementary material for: Value-conscious leadership actions in developing a health-promoting work environment
Source: Nurs Ethics. 2025 Sep 8;33(1):26–39. doi: 10.1177/09697330251366615 (PMC12907461; doi:10.1177/09697330251366615)
Supplement: Supplemental material - Value-conscious leadership actions in developing a health-promoting work environment [file sj-pdf-1-nej-10.1177_09697330251366615.pdf]

Value-conscious leadership actions in developing a health-promoting work environment

Supplemental file 1: Example list of codes, subthemes, themes, and overarching theme

Codes	sub- theme	Theme	Overaching theme
Equal treatment and fairness, openness in communication, trust and respect, consistency in leadership, I try to be fair and accommodating, it's important that you treat employees right, trust is what is strongest with me, show that you are following up on the small and big things, I'm a bit concerned with treating people equally, being generous with people when they're in trouble.	Fairness and individualized support	**Promoting justice by leading with equity and flexibility**	***Value-conscious leadership in action***
Flexibility and adaptation, individual approach, balancing between following rules and at the same time showing understanding for the employees' situations, creating a balance where both principles and individual needs are respected, there should be a balance, I have to adapt very much to them, at the same time harmonizing equal treatment and unique employee requirements	Balancing acts between upholding equal principles and individual prerequisites		
Rules and humanity, value-based leadership, the leader's role as a supporter, I have taken a lot of starting points in value-based leadership, it is important to have knowledge of rules and the working environment directive, and respecting humanity, I try to be professional, but also understanding, it is important that leaders talk together about fairness, we must have rules, but we must also be open about employees' challenges.	Balancing managerial regulations and humanistic values		

Role modeling through daily actions, accessibility and presence, honesty and openness, it's important to me that I get to greet each one, leading by example, demonstrating leadership values through actions, role modeling ethical behavior, open door policy, embodying trust, respect, and integrity in daily actions, showing consistency between words and actions, I'm happy that I'm in my department observing things. there should not be a distance between manager and employee.	Acting as role models: Embodying leadership values through daily actions	**Promoting relationships by cultivating an inclusive community**	
Openness and tolerance, respect for diversity, support groups, and debriefing, inclusive environment, we have worked a lot on having a lot of room to ask questions, it is allowed to be different, it is allowed to have different beliefs than you have, we have an open door policy, it is important to work to create community, it is allowed to have a hard time, and it is allowed to talk about it. creating safe spaces for open dialogue, promoting openness and mutual understanding.	Creating supportive spaces rooted in openness, tolerance, and respect for diversity		
Social gatherings, building team bonds through social activities, fostering friendships outside of work, encouraging social connections, creating a sense of belonging, community and unity, we have our own social committee that arranges social gatherings, we have joint lunches that we arrange together, inclusive activities, , we have several rounds where people have taken	The importance of social activities, friendship, and community		

home to each other and cooked together, it is important to have humor.			
Valuing employees' expertise, encouraging independent decision-making and influence, supporting autonomy in role and decision-making, ownership and participation, trust and empowerment, inclusive decision making, we have a system for influence, we have listened to them and acted on their suggestions.	Recognizing employees' abilities and fostering autonomy and influence.	**Respecting employees by promoting their empowerment and autonomy**	
Individual value and uniqueness, valuing differences in background, experience and perspectives, recognizing each employee as a unique and valuable part of the team, empowering through recognition, valuing individual contributions, fostering independence and empowering employees through trust and responsibility, fostering mutual respect in the workplace.	Empowering and appreciating employees as unique and valuable individuals		
empowering employees with greater responsibility, forstering self-confidence, we call it improvement suggestions instead of critique, responsibility and ownership, support and follow-up, confidence in own decisions, encouraging employees to take responsibility for their work and decisions, providing employees with support and follow-up to strengthen their sense of mastery, we have a quality group where employees can submit improvement suggestions.	Encouraging critical thinking and taking responsibility		
Individual preferences and interests, adaptation of work tasks, flexibility in work tasks, grouping based on interests, we have different fields of	Assessing learning needs and facilitating reflection groups based on shared interests	**Facilitating professional growth by promoting a learning environment**	

interest, commitment to specific tasks, high focus on each individual, personality type, we have divided into different groups, mapping of competence, individual preferences and interests, adaptation of work tasks, We have a group responsible for medical devices and it works well,			
Optimal use of competence, right competence in the right place, competence plans, task sharing based on competence, clarity in roles and responsibilities, defined expectations, task and responsibility sharing, work list, clear communication of expectations.	The right competence in the right place- role clarity and task sharing		
Mentor program for new employees, professional development through training, follow-up and support for new employees, we have professional days in our rotation, structured training plan, professional guidance and support, supporting continuous professional development, providing professional growth opportunities, training and supporting professional development.	Facilitating a professional development nurse and a mentorship program		
